# Gao-Zi-Yao improves learning and memory function in old spontaneous hypertensive rats

**DOI:** 10.1186/s12906-022-03630-0

**Published:** 2022-05-28

**Authors:** Meng-Xiao Han, Wen-Yi Jiang, Yan Jiang, Lin-Hui Wang, Rong Xue, Guo-Xing Zhang, Jing-Wei Chen

**Affiliations:** 1 Department of Physiology, Medical College of Soochow University, 199 Ren-Ai Road, Dushu Lake Campus, Suzhou Industrial Park, 215123 Suzhou, People’s Republic of China; 2 Suzhou Key Laboratory of Drug Research for Prevention and Treatment of Hyperlipidemic Diseases, Medical College of Soochow University, 199 Ren-Ai Road, Dushu Lake Campus, Suzhou Industrial Park, Suzhou, 215123 People’s Republic of China; 3 Department of Internal Medicine, the Affiliated Suzhou Chinese Traditional Medicine Hospital, Nanjing University of Chinese Medicine, 18 Yang-Su Road, Suzhou, 215003 People’s Republic of China

**Keywords:** Gao-Zi-Yao (oral herb medicine paste), Spontaneous hypertensive rats (SHR), Learning and memory

## Abstract

**Aims:**

Gao-Zi-Yao has long been a unique way for treating various diseases. The present study is to explore the effect of Gao-Zi-Yao on learning and memory function in old spontaneous hypertensive rats (SHR) and its possible mechanism.

**Method:**

Male old SHR were received different doses of Gao-Zi-Yao for 4 weeks. Systolic blood pressure (SBP) and heart rate were monitored. Serum levels of nitric oxide (NO), interleukin (IL)-1β, IL-2, and tumor necrotic factor (TNF)-α were measured. Morris water maze was performed to test the learning and memory function of the rats. Number of neurons in hippocampus was counted by Nissl staining. Western blot was applied to detect the expressions of learning and memory function related proteins, N-methyl-d-aspartate receptor 2B (NMDAR 2B), glutamate receptor 1 (GluR1), phosphorylated-calmodulin-dependent protein kinase II (p-CaMK II), and phosphorylated-cAMP responsive element-binding protein (p-CREB) in rat hippocampus.

**Results:**

Data showed that Gao-Zi-Yao reduced SBP in old SHR, elevated NO level, and suppressed levels of IL-1β, IL-2, TNF-α. The results of Morris water maze experiment showed that Gao-Zi-Yao dose-dependently improved learning and memory function. Number of neurons in the hippocampal dentate gyrus (DG) region of the old SHR was increased by Gao-Zi-Yao treatment. In addition, Gao-Zi-Yao elevated the protein expressions of NMDAR 2B, GluR1, p-CaMK II, and p-CREB in hippocampus.

**Conclusion:**

Gao-Zi-Yao decreases SBP and improves the learning and memory function of the old SHR by regulation of oxidative stress, inflammatory factors and neuron number in hippocampal DG area and the expression of learning and memory function related proteins.

**Supplementary Information:**

The online version contains supplementary material available at 10.1186/s12906-022-03630-0.

## Introduction

Aging has been demonstrated to be associated with a decline in cognitive abilities in the domains of perception, attention and working memory [1–3]. Aging is suggested to play a pivotal role in the pathogenesis and progression of one of the most dramatic age-related diseases, dementia (Alzheimer’s disease, AD) via oxidative stress signal pathway [4, 5]. AD is the most common type of dementia and typically manifests through a progressive loss of episodic memory and cognitive function, subsequently causing language and visuospatial skills deficiencies, which are often accompanied by behavioral disorders such as apathy, aggressiveness and depression [6, 7]. In addition, it has been recognized that hypertension is one of the major risk factors of dementia [8]. Hypertensive challenge increases the expression of the receptor for advanced glycated end products, leading to beta-amyloid (Aβ) deposition (one of the pathogenesis process of AD) and learning impairment [9, 10]. Due to complexity of aging induced dementia combined with hypertension, it is still no satisfactory strategy for AD patients with hypertension in clinic.

Traditional Chinese medicine (TCM) has more than 2,000 years of history and has gained widespread clinical applications. Effects of TCM on anti-aging have been widely recognized and have been well reviewed [11]. Clinical trials in China have demonstrated that TCM enhances adult hippocampal neurogenesis through activating the multi-signal pathways [12]. Randomized clinical trials studies in Europe also have demonstrated the improvement of cognitive decline of AD patients by TCM therapy [13]. In addition, TCM has been demonstrated to effectively lower blood pressure in patients [14]. Randomized controlled trial also demonstrated the certain antihypertensive effects and a good safety profile of TCM, and may likely serve as an alternative approach for hypertension patients [15].

Gaofang, one of oral herb medicine pastes, is a classic form of traditional Chinese medicine. Gao means herbal paste and fang is short for prescription. Gao-Zi-Yao refers to the oral herb paste prepared according to the special prescription, is made from several herbs, and is effective in strengthening immunity, preventing diseases, and nourishing health. Gao-Zi-Yao has been applied for treatment of many diseases such as sub-healthy condition, anorexia, anemia, chronic cough, menstrual disorder and so on for two thousand years. The present formula of Gao-Zi-Yao (Table 1) is modified from the practitioner of Wu Therapy of TCM, Tian-shi Ye (1666–1745, Qing Dynasty). According to exiting literatures, herbs included in the present formula have the following effects: antioxidant and neuroprotective activities of *Fructus Corni* was observed [16], *Semen Cuscutae* has been demonstrated to regulate immune system [17], Inflammatory response could be regulated by *Rhizoma Gastrodiae, Radix Cyathulae, Rhizoma Atractylodis Macrocephalae*, and *Fructus Amomi Villosi* [18–21], *Fructus Schisandrae Chinensis* shows anti-aging effects [22], *Semen Ziziphi Spinosae* was proved to ameliorate neuronal disorders [23], chronic fatigue syndrome could be improved by *Radix Pseudostellariae* [24], *plunus mume* exerts inhibitory effects of constituents on aldose reductase [25], *Crocus sativus* reduces oxidative stress [26], *Panax Notoginseng* has certainty effects on anti-aging and aging-related diseases [27], *Rhizome Pinelliae Preparata* promoted sleep by increasing the number of rapid eyes movement (REM) sleep episodes [28], *Alismatis Rhizoma* has diuretic, antimetabolic disorder, hepatoprotective, immunomodulatory, antiosteoporotic, anti-inflammatory, antitumor, antibacterial, and antiviral activities [29], *Radix Ophiopogonis* has anti-chronic inflammatory effect on senescent cells [30], *Coptidis Rhizoma*, combined with *Rhei Rhizoma, Scutellariae Radix*, shows antihypertensive effect [31], *Colla Corii Asini* improves antioxidant and antiproliferative activities [32]. *Colla Carapacis et Plastri Testudinis* contains various amino-acids and metal elements [33]. Yuan et al*.* observed catalpol, an active ingredient of *Rehmanniae radix preparata*, which is the most frequently used Chinese medicinal herb effectively ameliorate hyperactive and impulsive behavior, improve spatial learning and memory in SHR [34]. Recently, by applying the prescription, we demonstrated Gao-Zi-Yao exerts antihypertensive and anti-cardiovascular-remodeling effects in elderly SHR [35]. Hypertension can cause small vascular damage and partial white matter degeneration in the brain, SHR showed cognitive impairment with increasing age [36]. However, the effect of the present Gao-Zi-Yao on learning and memory function in old hypertension model is still unknown.

**Table 1:** Composition of Gao-Zi-Yao

	Latin name	English name	Amount (g)	place of origin
Shan Zhu Yu	*Cornus officinalis* Siebold & Zucc	Fructus Corni	300	Zhejiang, China
Tu Si Zi	*Cuscuta chinensis* Lam	Semen Cuscutae	100	Jiangsu, China
Tian Ma	*Gastrodia elata* Blume	Rhizoma Gastrodiae	150	Yunnan, China
Chuan Niu Xi	*Cyathula officinalis* K.C.Kuan	Radix Cyathulae	150	Sichuan, China
Bai Zhu	*Atractylis macrocephala* (Koidz.) Hand-Mazz	Rhizoma Atractylodis Macrocephalae	300	Jiangsu, China
Sha Ren	*Amomum villosum* Lour	Fructus Amomi Villosi	50	Yunnan, China
Wu Wei Zi	*Schisandra chinensis* (Turcz.) Baill	Fructus Schisandrae Chinensis	150	Hebei, China
Suan Zao Ren	*Ziziphus jujuba var. spinosa* (Bunge) Hu ex H.F.Chow	Semen Ziziphi Spinosae	150	Jiangsu, China
Tai Zi Shen	*Pseudostellaria heterophylla* (Miq.) Pax	Radix Pseudostellariae	150	Jiangsu, China
Lu E Mei	*Prunus mume* (Siebold) Siebold & Zucc	Prunus Mume	100	Jiangsu, China
Zang Hong Hua	*Crocus sativus* L	Crocus Sativus	1	Xizhan, China
San Qi	*Panax pseudoginseng var. notoginseng* (Burkill) G.Hoo & C.L.Tseng	Panax Notoginseng	50	Yunnan, China
Jiang Ban Xia	*Pinellia ternata* (Thunb.) Makino	Rhizome Pinelliae Preparata	150	Jiangsu, China
Ze Xie	*Alisma plantago-aquatica* subsp. *orientale* (Sam.) Sam	Alismatis Rhizoma	150	Fujian, China
Mai Dong	*Ophiopogon japonicus* (Thunb.) Ker Gawl	Radix Ophiopogonis	150	Jiangsu, China
Huan Lian	*Coptis chinensis* Franch	Rhizoma Coptidis	60	Jiangsu, China
E Jiao	Colla Corii Asini	Colla Corii Asini	300	Shandong, China
Gui Jia Jiao	Colla Carapacis et Plastri Testudinis	Colla Carapacis et Plastri Testudinis	200	Jiangsu, China

In the present observation, we applied Gao-Zi-Yao on old spontaneous hypertensive rats (SHR) to investigate its anti-hypertensive neuroprotective effects, and to explore the possible mechanisms involved in.

## Materials and methods

### Preparation of oral her medicine paste

**Fig. 1:**
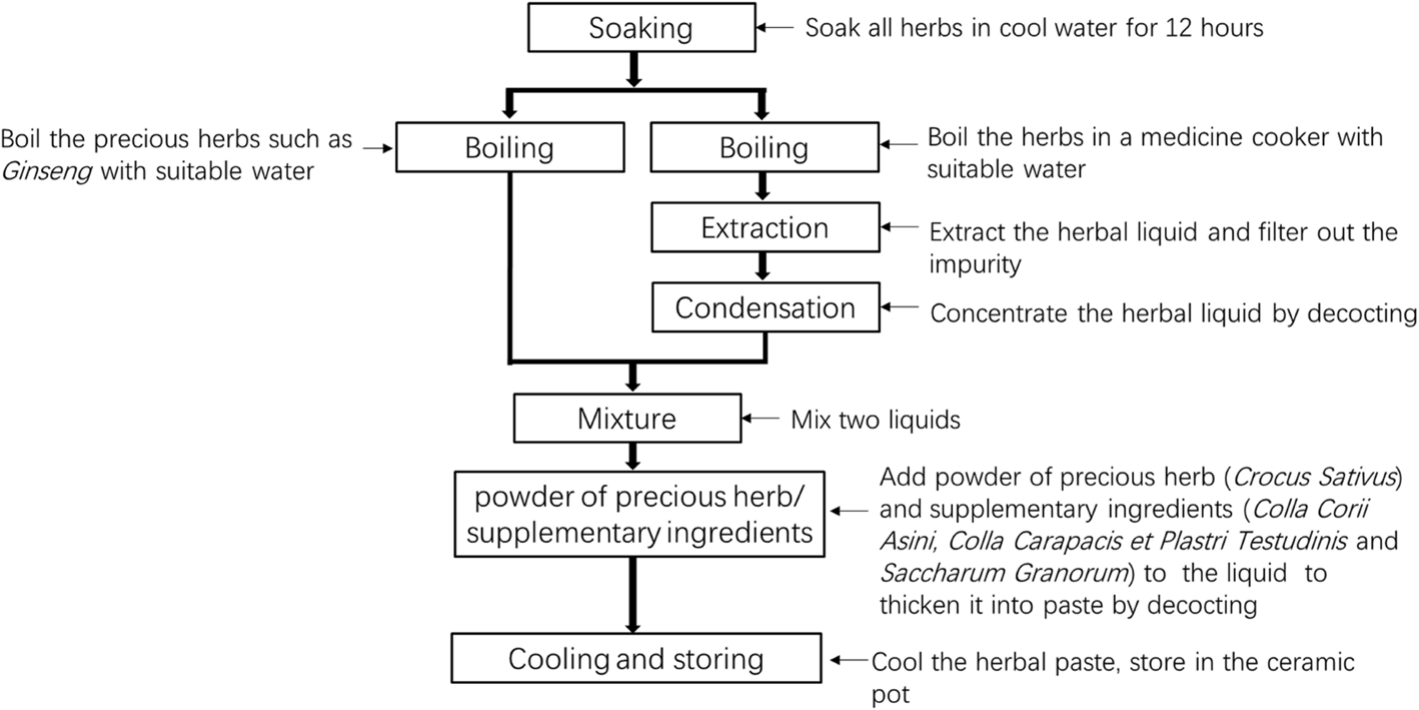
Production of process of Gao-Zi-Yao

Gao-Zi-Yao was prepared from bellowing listed raw materials (Table 1) purchased from Suzhou Chinese Traditional Medicine Hospital (Suzhou, China) and were identified by Duo-Rong Sheng, a pharmacist of traditional Chinese medicine in Suzhou Chinese Traditional Medicine Hospital. All voucher specimens are deposited in the herbarium center of Suzhou Chinese Traditional Medicine Hospital (deposition number is unavailable)**.** All raw materials were soaked for 24 h, and then boiled for three times, condensed boiled liquid of three times to about 200 mL, mixed with dissolved *Colla Corii Asini*, *Colla Carapacis et Plastri Testudinis* glue, and *Saccharum Granorum* to form paste for further application. Production process is described in Fig. 1. All procedures are according to the standard of Chinese Pharmacopoeia (2010 edition) [35].

### Experimental animals

Twelve-month-old and eight-week-old male spontaneous hypertensive rats (SHR) were purchased from Nanjing Animal Model Center. In this model, hypertension starts developing by 4 months of age with the appearance of hypertension-related changes in the brain microvasculature by 6 months of age [37]. Rats were housed under optimal conditions with standard hygiene, kept at a temperature of 25 °C with a 12/12 light/dark cycle, fed with standard rat chow and water ad libitum. All procedures were approved by and performed according to guidelines for the care and use of animals established by Soochow University, which is consistent with our previous report [35]. The experiments were performed in according with the National Institutes of Health Guidelines for the Use of Laboratory Animals (NIH, publication number 85–23, revised 1996). The present study is reported in accordance with ARRIVE guidelines.

Eight-week-old SHR was applied for aging control (SHR-Young, *n* = 10). Twelve-month-old rats were treated with or without Gao-Zi-Yao for distilled water (Old SHR-C, *n* = 10), low dose (Old SHR-L, *n* = 10), medium dose (Old SHR-M, *n* = 10), high dose (Old SHR-H, *n* = 10) by gastric feeding. Dosages of low, medium, and high of Gao-Zi-Yao equal to 1/2-, 1-, threefold dosages for clinical patient application calculated according to body surface area. Gao-Zi-Yao was dissolved in 5 mL distilled water before application and was administrated one time dairy for 4 weeks. Blood pressure and heart rate were monitored every week by tail-cuff method, and body weight was also monitored every week as previous our observation [35]. After 4 weeks, Morris water maze experiment was performed, and rats were sacrificed under anesthesia by sodium pentobarbital (50 mg/kg i.p.), hippocampus tissue was collected for further analysis. All experimental protocols were approved by ethics licensing committee of Soochow University.

### Morris water maze experiment

Morris water maze experiment (BW-MWM101, Shanghai Bio-will Co., Ltd., Shanghai, China) was performed as described previously with some modifications [38]. Test was planned for 6 days, day 1 to 5 is for the training period, day 6 is for testing results. Rats were received training for 2 min dairy, recording time for boarding on platform as the latency time. On day 6 the platform was removed. Rats were put into the farthest distance quadrant and the swimming trajectory was recorded for 2 min. Parameters for learning and memory function were calculated by the software.

### Measurement of serum levels of NO and inflammatory factors by ELISA

Serum levels of NO (Catalog No: S0023), IL-1β (Catalogue number: A301BH80153), IL-2 (Catalogue number: A31038348), IL-6 (Catalogue number: A30681042), and TNF-α (Catalogue number: A38280855) were measured using commercially available ELISA kits (Biotechnology Co., Ltd. Shanghai enzyme research. Shanghai, China). All steps were performed according to the manufacturer’s instructions.

### Nissl staining

Rat hippocampus tissue was isolated, fixed, paraffin embedded, then incubated in 1% toluidine blue staining solution for 5–10 min at room temperature. Then the sections were rinsed in distilled water, soaked in 95% ethanol for 5–30 min and dehydrated in 100% ethanol. After dehydration slice was placed in xylene and cover-slipped using resin medium. The number of neurons in the CA1, CA2 and dentate gyrus (DG) regions of the hippocampus were observed and analyzed using the ImageJ analysis program.

### Western blot for learning and memory related proteins

Western blot for learning and memory related proteins was carried out as described in our previous report with some modification [39]. Hippocampus tissues were homogenized with RIPA buffer (50 mm Tris, 150 mM NaCl, 1% Triton-X-100, pH 7.0) containing phenylmenthanesulfonyl fluoride (R&D Systems Inc., Minneapolis, US). Homogenates were centrifuged at 12,000 × g for 10 min at 4 °C. Cell protein were separated by SDS-PAGE and transferred to PVDF membranes (Hybond TM-ECL; Amersham Pharmacia Biotech, Inc.). The membranes were blocked in 5% nonfat milk in PBS and 0.1% Tween-20 at room temperature. The blots were then incubated with primary antibody: Anti-glutamate receptor 1 (1:1000, abcam, Inc., Catalog No: ab183797), Anti-NMDAR2B antibody (1:1000, abcam, Inc., Catalog No: ab28373), Anti-phospho-CaMKII antibody (1:1000, abcam, Inc., Catalog No: ab171095), Anti-phospho-CREB antibody (1:1000, abcam, Inc., Catalog No: 32096) or anti-GAPDH (Santa Cruz Biotech, Inc., Catalog No: sc-47724). Next, membranes were incubated for 1 h with a secondary antibody (HRP-conjugated anti-rabbit Ig-G, 1:2000, Abcam, Inc. Catalog No: ab205718). Membranes were then three-time washed for 15 min using TBS-T to remove excess antibody before incubation for 1 min with chemiluminescent reagents (ECL, R&D Systems Inc., Minneapolis, MN, USA). Further, immunoreactive bands were detected by an electrophoresis gel analysis system (GL2200 Pro, Crestream Inc. USA). The intensity of the bands was analyzed by Image J software. The quantity of target proteins was normalized by GAPDH expression [39].

### Statistical analysis

The SPSS 18.0 software was used for statistical analysis. Data are presented as the mean ± S.E.M. Grouped data were analyzed using a one-way analysis of variance followed by the Student–Newman–Keuls test. A *P* value < 0.05 was considered as statistically significant as our previous report [35].

## Results

### Systolic blood pressure (SBP), heart rate and body weight between young SHR and old SHR

**Fig. 2:**
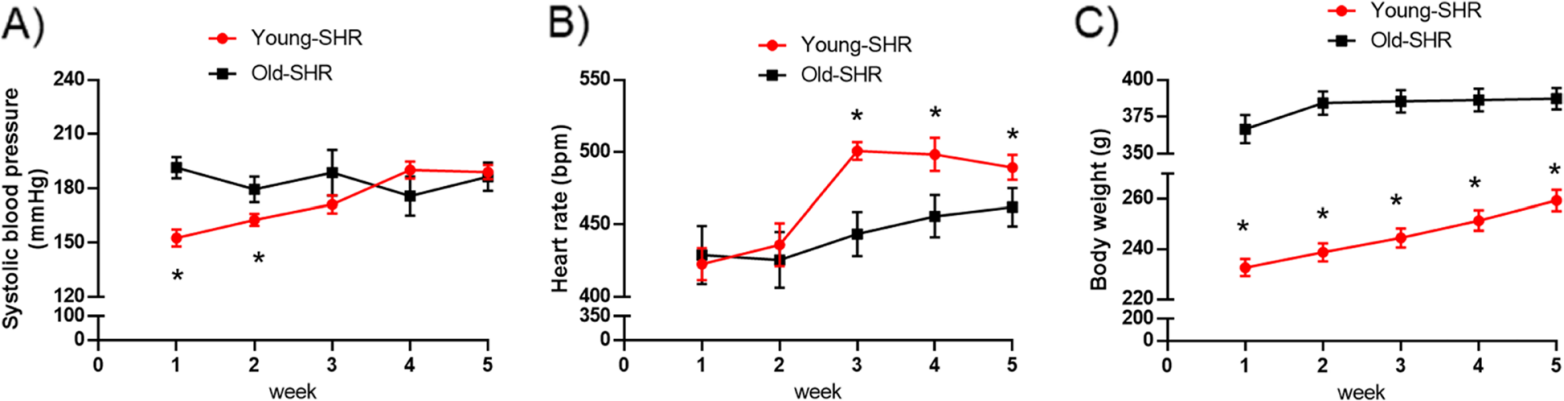
Systolic blood pressure (SBP), heart rate and body weight in each group. **A** SBP in each group, **B** Heart rate in each group, **C** body weight in each group. *n* = 10, *P < 0.05 young SHR group compared with old SHR group

Yong SHR showed lower SBP at the first two weeks compared with old SHR, then increased to no difference between two groups at the later three weeks (Fig. 2A). On the contrary, heart rate was no difference between two groups at the first two weeks, then young SHR increased markedly compared with old SHR at the later three weeks (Fig. 2B). Young SHR showed lower body weight compared with old SHR during the whole observing period (Fig. 2C).

### Morris water maze parameters between young SHR and old SHR

**Fig. 3:**
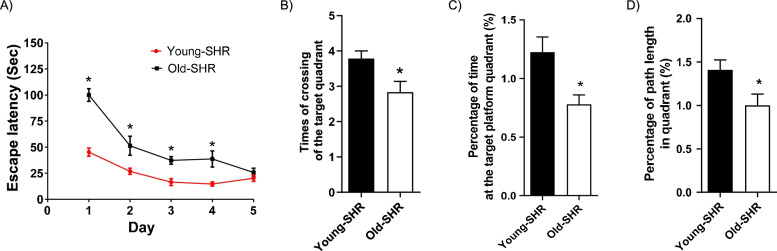
Morris water maze parameters. **A** Escape latency in each group, **B** Times of crossing the target quadrant, **C** Percentage of time at the target platform quadrant, **D** Percentage of path length in quadrant. *n* = 10. *P < 0.05 young SHR group compared with young SHR group

To determine hippocampal dependent learning and memory, Morris water maze experiment was performed. Old SHR showed longer escape latency at the first four days compared with young SHR (Fig. 3A). Times of crossing the target quadrant was less in old SHR than that in young SHR (Fig. 3B). Percentage of time at the target platform quadrant was shorter in old SHR compared with young SHR (Fig. 3C). Percentage of path length in quadrant was also shorter in old SHR compared with old SHR (Fig. 3D). These results suggest old SHR has impairment in recognitive function.

### Number of neurons in hippocampus between young SHR and old SHR

**Fig. 4:**
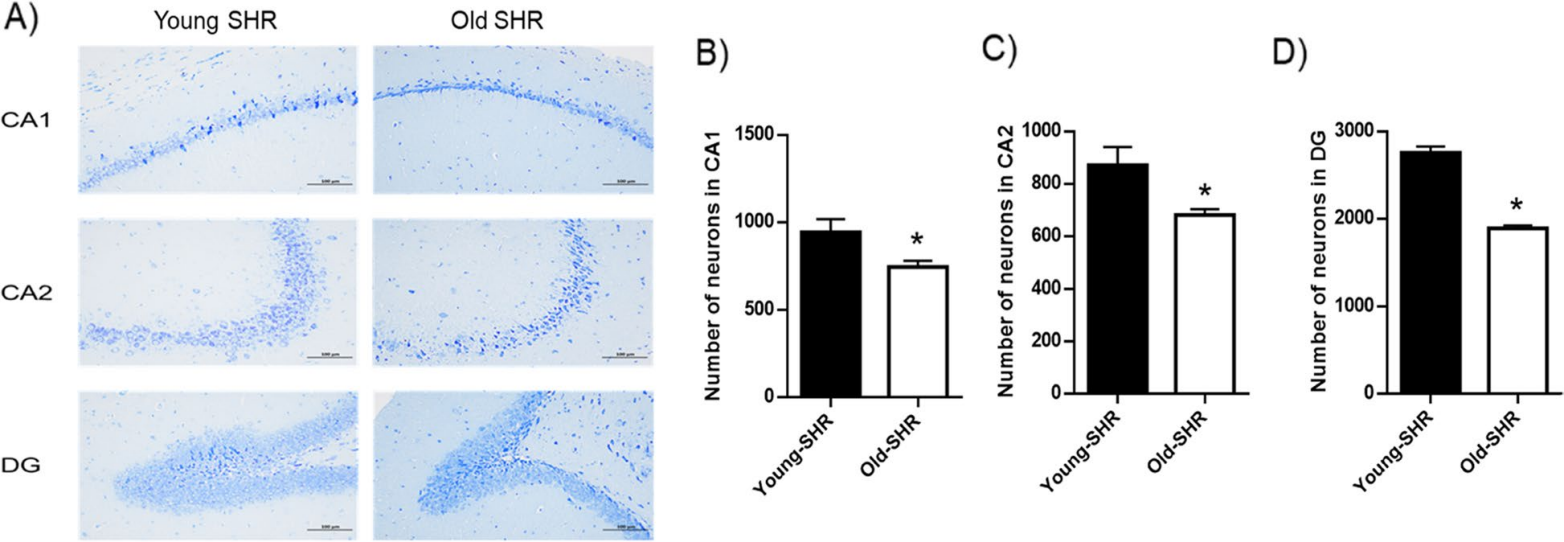
Nissl staining results in each group. **A** Representative Nissl-staining of CA1, CA2 and DG area in each group, **B** Number of neurons at CA1 area in each group, **C** Number of neurons at CA2 area in each group, **D** Number of neurons at DG area in each group. *n* = 6. *P < 0.05 young SHR group compared with young SHR group

Number of neurons in different areas of hippocampus was counted by Nissl staining. Results showed that there are a smaller number of neurons in CA1, CA2 and DG region of old SHR compared with young SHR (Fig. 4).

### Learning and memory related protein expressions at hippocampus between young SHR and old SHR

**Fig. 5:**
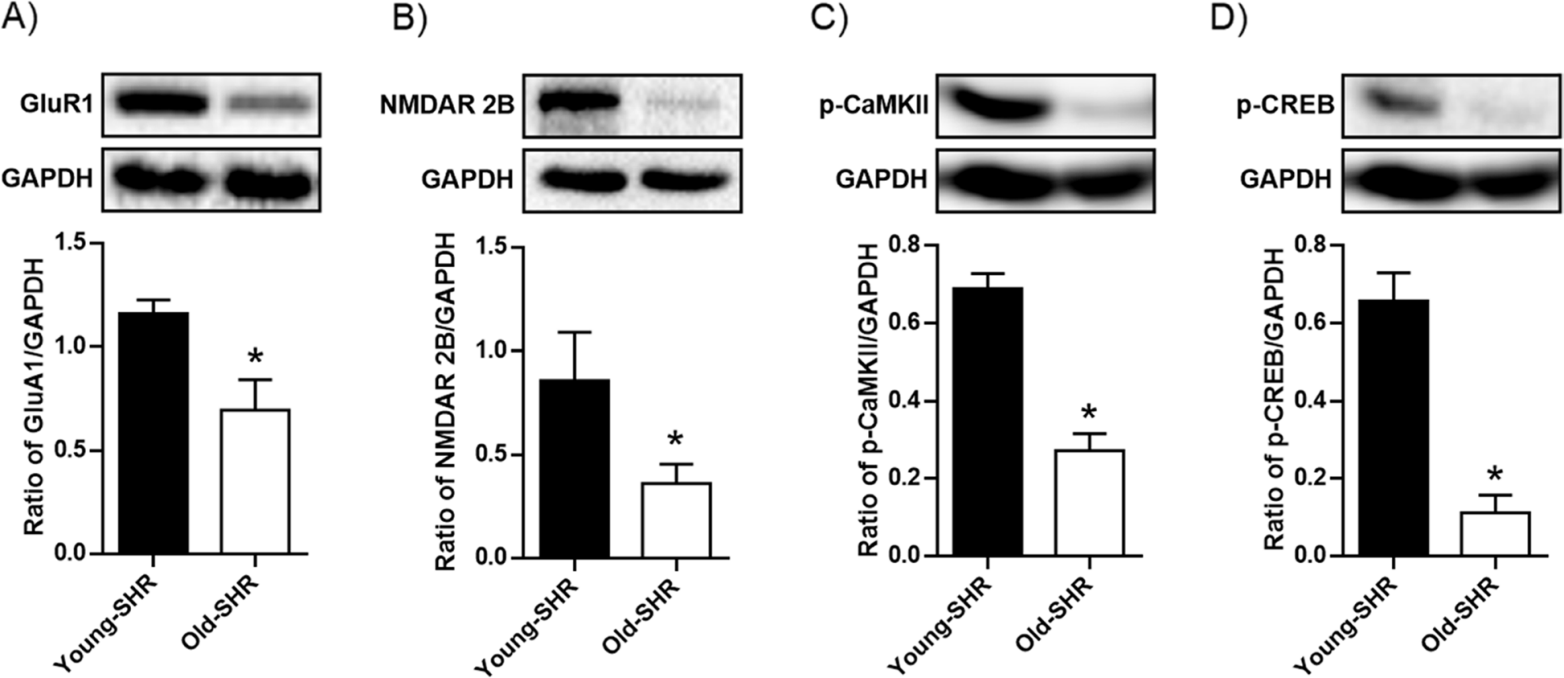
Protein expression of GluR1, NMDAR 2B, phosphorylated-CaMK II, and phosphorylated-CREB in each group. **A** Protein expression of GluR1 in each group, upper: representative blots of GluR1 and GAPDH, down: densitometry analysis of GluR1 and GAPDH. **B** Protein expression of NMDAR 2B in each group, upper: representative blots of NMDAR 2B and GAPDH, down: densitometry analysis of NMDAR 2B and GAPDH. **C** Protein expression of phosphorylated-CaMK II (p-CaMK II) in each group, upper: representative blots of p-CaMK II and GAPDH, down: densitometry analysis of p-CaMK II and GAPDH. **D** Protein expression of phosphorylated-CREB (p-CREB) in each group, upper: representative blots of p-CREB and GAPDH, down: densitometry analysis of p-CREB and GAPDH. *P < 0.05 young SHR group compared with young SHR group

Western blot results showed that expression of learning and memory related proteins (GluR1, NMDAR 2B, phosphorylated-CaMK II, and phosphorylated-CREB) in old SHR hippocampus were lower than that in young SHR hippocampus (Fig. 5).

### Effect of Gao-Zi-Yao on SBP, heart rate and body weight in old SHR

**Fig. 6:**
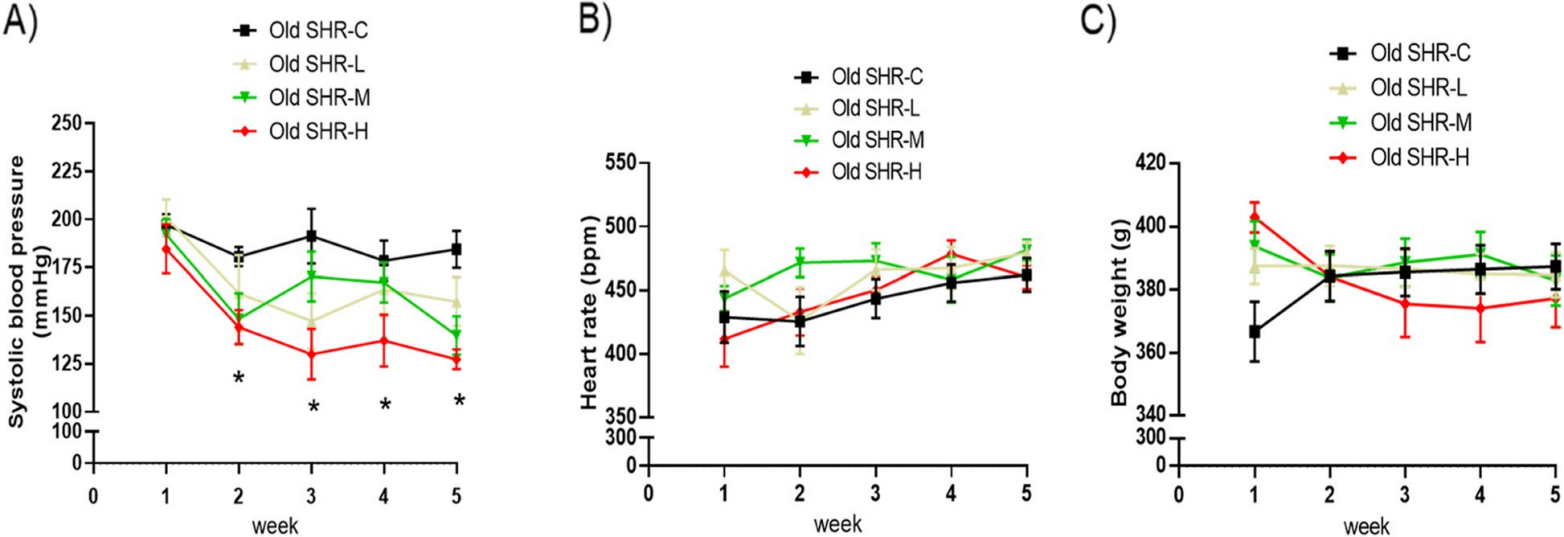
Systolic blood pressure (SBP), heart rate and body weight in each group. **A** SBP in each group, **B** heart rate in each group, **C** body weight in each group. Old SHR-C indicates old SHR control group (*n* = 10), Old SHR-L indicates old SHR treated with low dosage of Gao-Zi-Yao (*n* = 10), Old SHR-M indicates old SHR treated with medium dosage of Gao-Zi-Yao (*n* = 10), Old SHR-H indicates old SHR treated with high dosage of Gao-Zi-Yao (*n* = 10). *P < 0.05 old SHR-L, old SHR-M, and old SHR-H compared with old SHR control group

Effect of Gao-Zi-Yao on SBP was analyzed. Treatment with low dosage of Gao-Zi-Yao markedly decreased SBP in old SHR from the second week to the fourth week compared with control old SHR group, medium and high dosages of Gao-Zi-Yao markedly decreased SBP in old SHR from the first week to the fourth week compared with control old SHR (Fig. 6A). These results indicate Gao-Zi-Yao exerts anti-hypertensive effect in old SHR.

We also compared the data of effects of Gao-Zi-Yao on heart rate and body weight. Our data demonstrated that there was no significant difference of heart rate and body weight among all groups during the whole observation period (Fig. 6B, C).

### Effect of Gao-Zi-Yao on serum levels of NO, IL-1β, IL-2 and TNF-α in old SHR

**Fig. 7:**
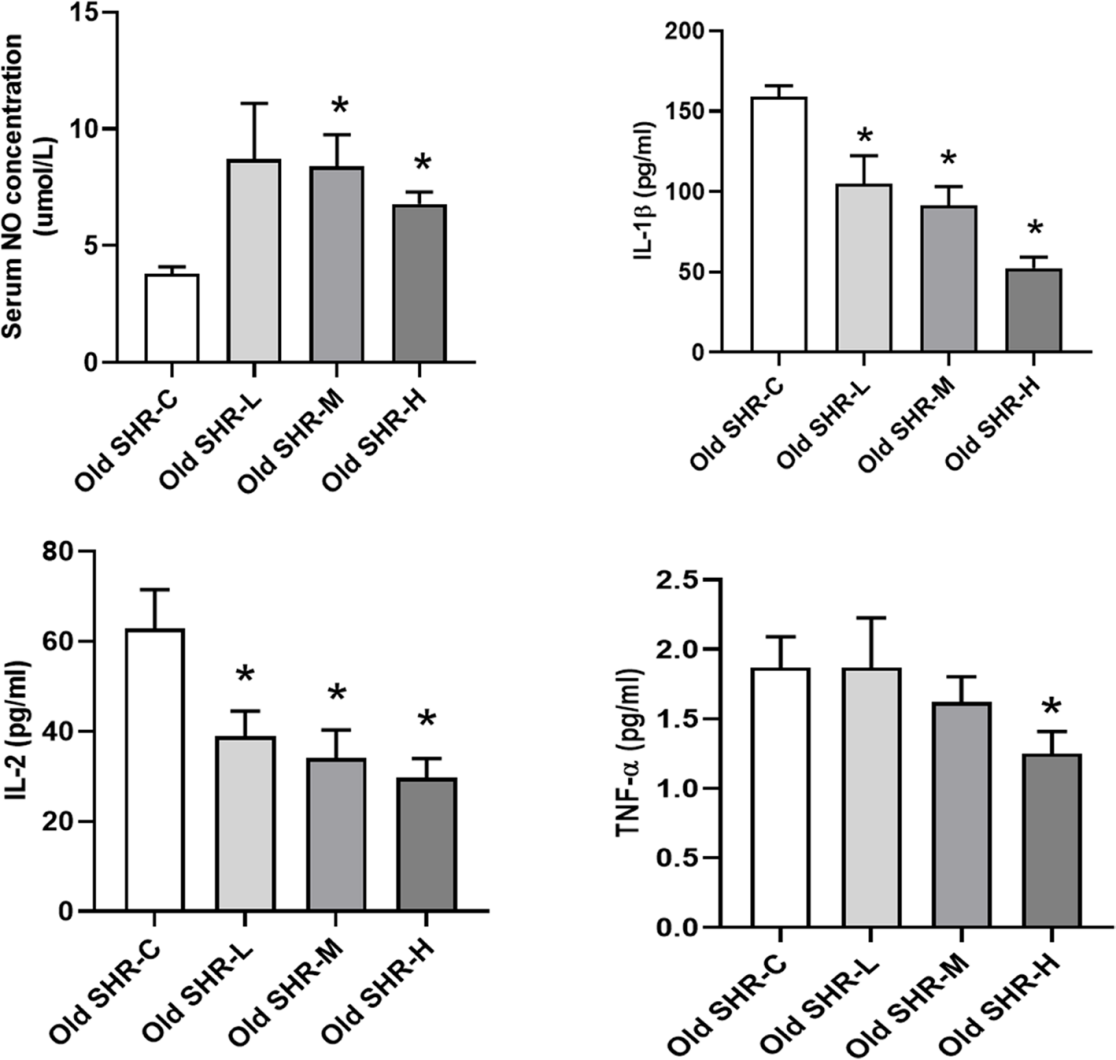
Serum contents of NO, inflammatory factors in each group. **A** Serum NO content, *P < 0.05 old SHR-M, and old SHR-H compared with old SHR control group. **B** Serum content of IL-1β, *P < 0.05 old SHR-L, old SHR-M, and old SHR-H compared with old SHR control group. **C** Serum content of IL-2, *P < 0.05 old SHR-L, old SHR-M, and old SHR-H compared with old SHR control group. **D** Serum content of TNF-α, *P < 0.05 old SHR-H compared with old SHR control group. Old SHR-C indicates old SHR control group (*n* = 5), Old SHR-L indicates old SHR treated with low dosage of Gao-Zi-Yao (*n* = 5), Old SHR-M indicates old SHR treated with medium dosage of Gao-Zi-Yao (*n* = 5), Old SHR-H indicates old SHR treated with high dosage of Gao-Zi-Yao (*n* = 5)

Treatment with medium and high dosages of GAO-ZI-YAO increased the serum NO levels in comparison with the levels in the SHR control group (Fig. 7A). Treatment with all tested dosages of GAO-ZI-YAO reduced the serum levels of IL-1β, IL-2 in comparison with the levels in the SHR control group (Fig. 7B, C); however, only the high dosage of GAO-ZI-YAO suppressed the serum levels of TNF-α (Fig. 7D). These results demonstrate that GAO-ZI-YAO could regulate oxidative stress and inflammation.

### Effect of Gao-Zi-Yao on Morris water maze parameters in old SHR

**Fig. 8:**
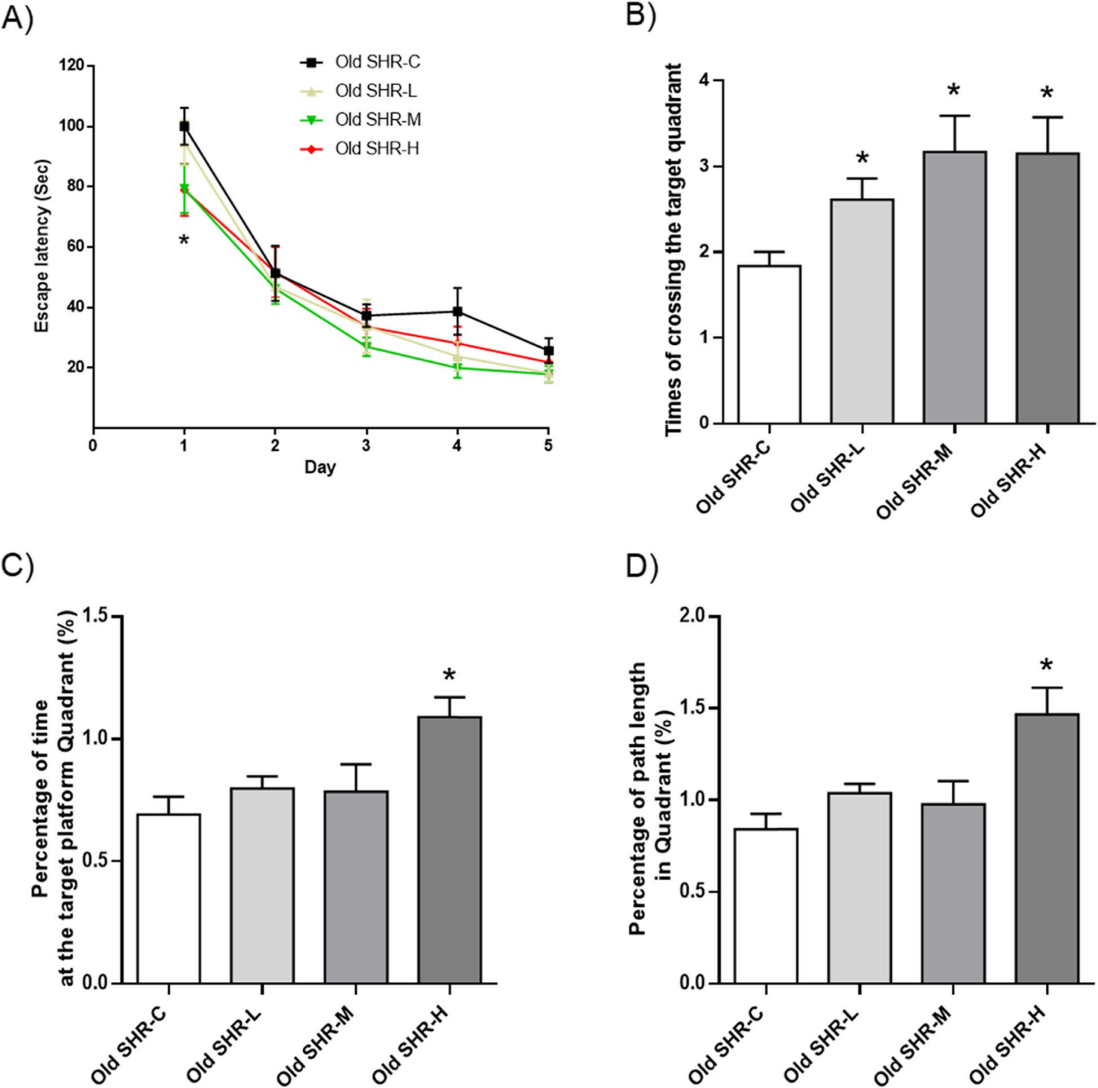
Morris water maze parameters. **A** Escape latency in each group, *P < 0.05 old SHR-M and old SHR-H compared with old SHR control group. **B** Times of crossing the target quadrant, *P < 0.05 old SHR-L, old SHR-M, and old SHR-H compared with old SHR control group. **C** Percentage of time at the target platform quadrant, *P < 0.05 old SHR-H compared with old SHR control group. **D** Percentage of path length in quadrant, *P < 0.05 old SHR-H compared with old SHR control group. Old SHR-C indicates old SHR control group (*n* = 10), Old SHR-L indicates old SHR treated with low dosage of Gao-Zi-Yao (*n* = 10), Old SHR-M indicates old SHR treated with medium dosage of Gao-Zi-Yao (*n* = 10), Old SHR-H indicates old SHR treated with high dosage of Gao-Zi-Yao (*n* = 10). *P < 0.05 compared with old SHR control group

Morris water maze data showed that both of medium and high dosages of Gao-Zi-Yao treatment significantly reduced escape latency time on the first training day, from the second day, the difference was not existed (Fig. 8A). Times of crossing the target quadrant was markedly increased in all dosage treated group on day 6 compared with control group (Fig. 8B). Only high dosage of Gao-Zi-Yao treatment markedly increased percentage of time at the target platform quadrant and percentage of path length in quadrant (Fig. 8C, D). These results suggest Gao-Zi-Yao could ameliorate the decline of learning and memory function in old SHR.

### Effect of Gao-Zi-Yao on number of neurons in hippocampus in old SHR

**Fig. 9:**
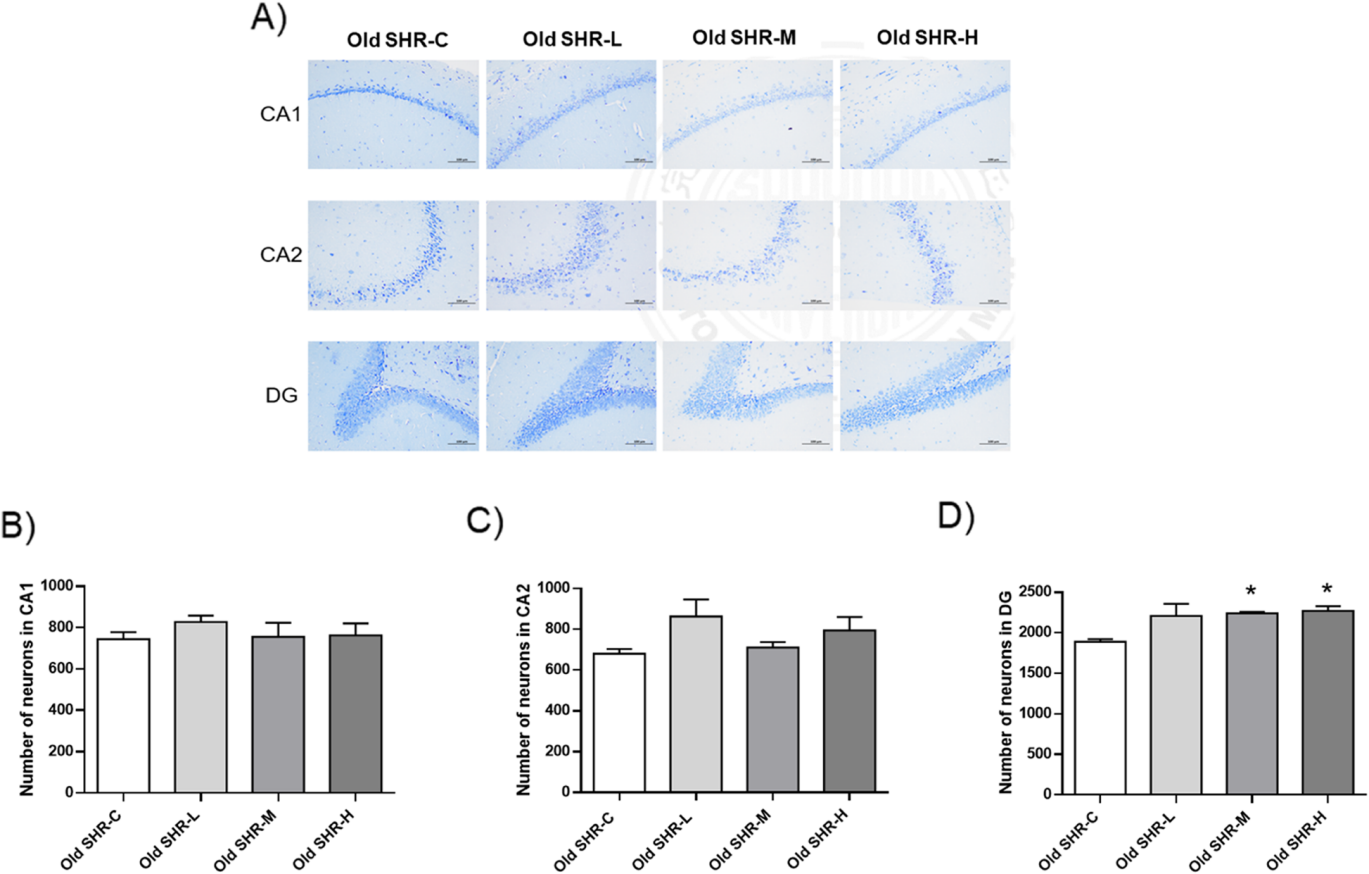
Nissl staining results in each group. **A** Representative Nissl-staining of CA1, CA2 and DG area in each group, **B** Number of neurons at CA1 area in each group, **C** Number of neurons at CA2 area in each group, **D** Number of neurons at DG area in each group. *n* = 6. Old SHR-C indicates old SHR control group, Old SHR-L indicates old SHR treated with low dosage of Gao-Zi-Yao, Old SHR-M indicates old SHR treated with medium dosage of Gao-Zi-Yao, Old SHR-H indicates old SHR treated with high dosage of Gao-Zi-Yao. *P < 0.05 old SHR-M, and old SHR-H compared with old SHR control group

Nissl staining was performed to demonstrate the number neurons at hippocampus (Fig. 9A). Results showed that treatment with Gao-Zi-Yao with all dosages had no marked effect on number of neurons in CA1 and CA2 areas in hippocampus (Fig. 9B, C). However, number of neurons in DG area was markedly increased by medium and high dosages of Gao-Zi-Yao treatment, but not the low dosage of Gao-Zi-Yao (Fig. 9D). These data suggest Gao-Zi-Yao could protect ageing related neuron loss in old SHR.

### Effect of Gao-Zi-Yao on expressions of learning and memory related protein at hippocampus in old SHR

**Fig. 10:**
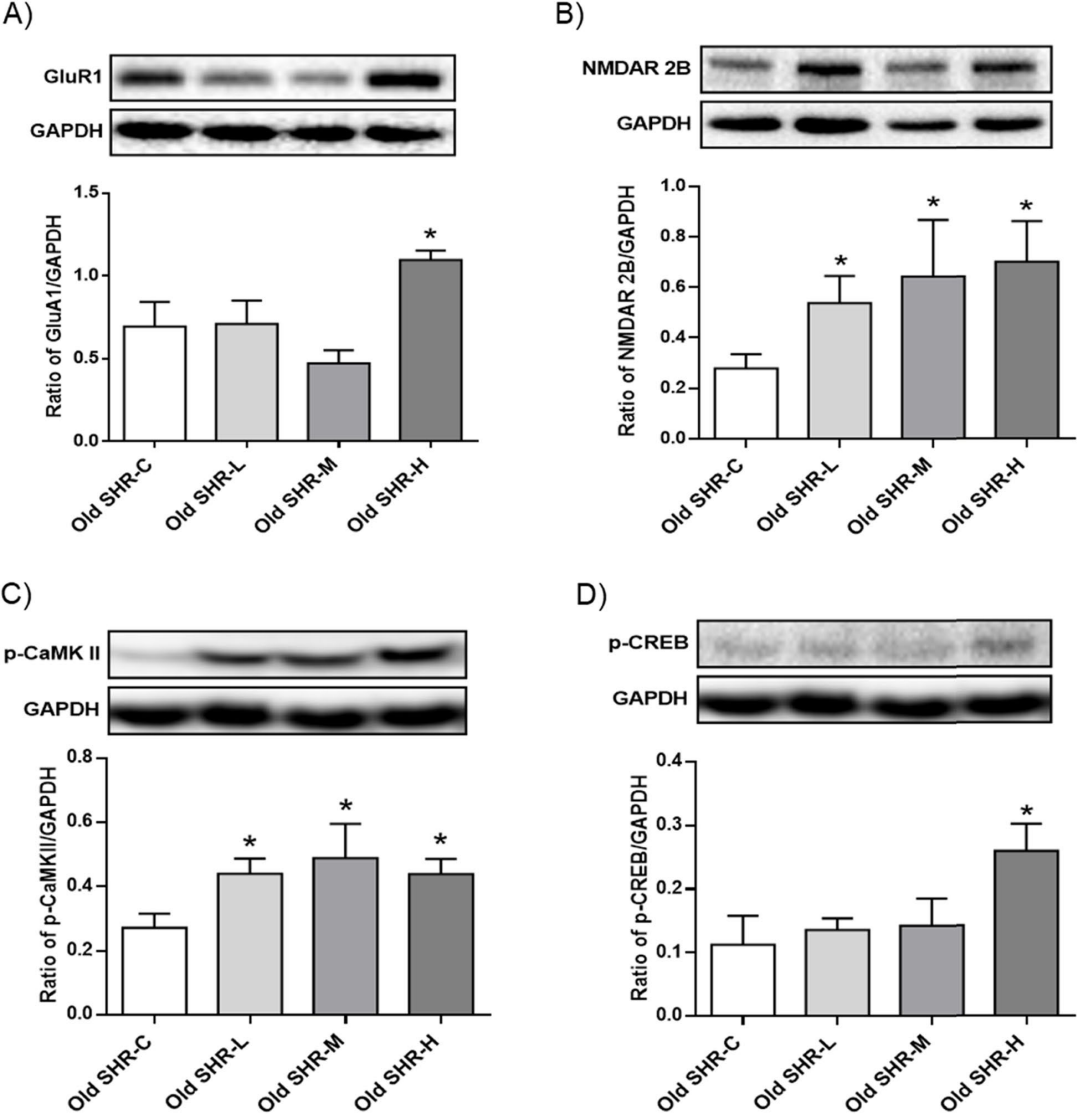
Protein expression of GluR1, NMDAR 2B, phosphorylated-CaMK II, and phosphorylated-CREB in each group. **A** Protein expression of GluR1 in each group, upper: representative blots of GluR1 and GAPDH, down: densitometry analysis of GluR1 and GAPDH. *P < 0.05 old SHR-H compared with old SHR control group. **B** Protein expression of NMDAR 2B in each group, upper: representative blots of NMDAR 2B and GAPDH, down: densitometry analysis of NMDAR 2B and GAPDH, *P < 0.05 old SHR-L, old SHR-M, and old SHR-H compared with old SHR control group. **C** Protein expression of phosphorylated-CaMK II (p-CaMK II) in each group, upper: representative blots of p-CaMK II and GAPDH, down: densitometry analysis of p-CaMK II and GAPDH. *P < 0.05 old SHR-L, old SHR-M, and old SHR-H compared with old SHR control group. **D** Protein expression of phosphorylated-CREB (p-CREB) in each group, upper: representative blots of p-CREB and GAPDH, down: densitometry analysis of p-CREB and GAPDH. *n* = 6. *P < 0.05 old SHR-H compared with old SHR control group. Old SHR-C indicates old SHR control group, Old SHR-L indicates old SHR treated with low dosage of Gao-Zi-Yao, Old SHR-M indicates old SHR treated with medium dosage of Gao-Zi-Yao, Old SHR-H indicates old SHR treated with high dosage of Gao-Zi-Yao

Western blot results showed that expression of GluR1 protein was increased by high dosage of Gao-Zi-Yao (Fig. 10A), NMDAR 2B protein expression was increased by all dosages of Gao-Zi-Yao (Fig. 10B), phosphorylated-CaMK II was increased by all dosages of Gao-Zi-Yao (Fig. 10C), and phosphorylated-CREB was increased by high dosage of Gao-Zi-Yao (Fig. 10D). These results suggest Gao-Zi-Yao might be to enhance synaptic plasticity by up-regulating synaptic plasticity related protein expressions.

## Discussion

In the present study, our observation reveals that Gao-Zi-Yao exerts anti-hypertensive and improves the learning and memory function of the old SHR, which may by regulation of oxidative stress, inflammatory factors, neuron number in hippocampal DG area and the expression of function of learning and memory related proteins.

Increasing evidence shows an important relationship between aging and hypertension and it was well reviewed [40]. Aging increases oxidative stress, which is one of the central factors as the development of hypertension. Existing data showed that antioxidant treatment to reduce oxidative stress prevents the age-related development of high blood pressure in SHR [41, 42]. Our present Gao-Zi-Yao contains several herbs possess antioxidant effect, and could regulate tissue oxidative stress, such as *Fructus Corni*, *Semen Cuscutae*, *Rhizoma Gastrodiae, Radix Cyathulae, Rhizoma Atractylodis Macrocephalae*, *Amomum villosum* and *Crocus sativus * [16–21, 26]. Our present data clearly showed that Gao-Zi-Yao could increase serum level of NO, reduce systolic blood pressure in old SHR, suggesting that anti-hypertensive property of GAO-Zi-Yao might be due to its antioxidant effect.

Although recent clinical trial demonstrated that only two-thirds of Alzheimer’s dementia cases are attributable to common age-related neuropathology [43], aging is generally accepted to be one of the main factors in the development of dementia [44, 45]. Age-dependent memory impairments animal model was reported [46]. Numerous evidence showed that there is a cognitive behavior decline in dementia animal models [47]. Meanwhile, hypertension has more recently been linked to Alzheimer disease-the major cause of dementia in older people, hypertension may also promote the neurodegenerative pathology underlying Alzheimer disease [48]. Recent clinical trials have also indicated that improved hypertension control reduces the risk for cognitive impairment and dementia [49]. In the present study, we observed there is also a decline of learning and memory function in old SHR, which was consistent with previous observation reported that working memory and learning were found to be impaired by aging [50]. In addition, it was reported that significantly suppressed Morris water maze performance was found in 23-wk SHR in comparison with age-matched SD rats [51]. Taking together, existing evidence strongly suggested that there is a decline of learning and memory function in old SHR. Aging combined with hypertension is critical factors for the development of dementia. Gao-Zi-Yao exerts its function may partially by its antihypertensive property. In addition, oxidative damage is a key component of Alzheimer disease aetiology and pathogenesis [52], Gao-Zi-Yao increases systemic NO level, which may also contribute to ameliorate dysfunction of cerebral system. There is a growing body of evidence that both local and systemic inflammation are important in dementia [53], our present data also demonstrated that Gao-Zi-Yao exerts regulation effects on systemic inflammation by decrease of IL-1β, IL-2 and. TNF-α, which may also play a role in the present animal model. Furthermore, pronounced age-related decline in the number of neurons was observed in animal [54], aging caused significant decrease of Nissl body amounts in hippocampal CA1 and CA3 regions in senescence-accelerated mice [55], in the present study, we also observed there is decline of neuron number in hippocampus in old SHR compared with young SHR, suggesting the impairment of learning and memory in old SHR might be due to the loss of neuron. There were significant impairments in long term potential in middle-aged rat hippocampus slices compared with that of young rat, and decrease of GluR1, NMDAR protein expression, suggesting impaired synaptic plasticity by aging [56]. The other markers of synaptic plasticity, calmodulin-dependent protein kinase II (CaMKII) and phosphorylated CaMKII, CREB, phosphorylated CREB, were also reported to be aging-related [57]. Our present study also demonstrated there is a lower level of synaptic plasticity related protein expressions (GluR1, NMDAR 2B, phosphorylated CaMKII, phosphorylated CREB) in old SHR, suggesting there is also a decline of synaptic plasticity, which might be contribute to the impairment of learning and memory function in old SHR.

Increasing evidence demonstrated that TCM therapy has potential effects on improvement of cognitive function [58]. Randomized controlled trials data supported the positive effects of TCM on age associated memory impairment [59]. Several clinical trials also demonstrated the effective outcome of TCM on enhancement of memory on ageing patients or healthy person [60, 61]. Several herbs included in the present prescription have been demonstrated to improve recognitive function. Loganin is a major iridoid glycoside obtained from *Corni fructus* enhances long-term potentiation and recovers scopolamine-induced learning and memory impairments [62]. *Semen Cuscutae* attenuate scopolamine-induced memory deficit in mice [63]. Gastrodin, an active component isolated from the *Rhizome Gastrodiae*, significantly improved memory impairments in the Morris water maze test in mice [64]. *Rhizoma Atractylodis Macrocephalae* contained prescription has a protective effect against ischemia-induced neuronal and cognitive impairments [65]. α-Isocubebenol and deoxyschisandrin are isolated from *Fructus Schisandrae Chinensis*, showed protective effects on cognitive impairment [66, 67]. *Semen Ziziphi Spinosae* ameliorates memory and learning performance in mice [23, 68]. MSS, a comprising mixture of maesil (*Prunus mume* Sieb. et Zucc) concentrate, disodium succinate and Span80 (3.6:4.6:1 ratio) showed a significant improvement of memory in rat [69]. *Crocus sativus* L. extracts antagonize memory impairments in different behavioural tasks in the rat [70]. *Panax Notoginseng* attenuates impairment of learning and memory in chronic stage ischemia–reperfusion injured rats [71] and in Aβ (1–42)-injected Rats [72]. *Radix Ophiopogonis*, component of Shengmaisan, improves learning and memory abilities of the rats [73]. Berberine, a natural isoquinoline alkaloid isolated from the *Rhizoma coptidis*, mitigates cognitive decline in an Alzheimer’s Disease mouse model [74, 75]. All above-mentioned literatures support that Gao-Zi-Yao might also improve learning and memory in old SHR. Our data clearly demonstrated that Gao-Zi-Yao treatment decreases systolic blood pressure, regulates oxidative stress and inflammation, improves learning and memory, up-regulates number of neurons and synaptic plasticity related protein expressions. These data strongly confirm that Gao-Zi-Yao could exert neuroprotective effects.

## Limitation

It should be noted that whether decrease of blood pressure contributes to the improvement of recognitive function was not included in the present observation. Since hypertension has been demonstrated to be an independent factor in the development of dementia, it is reasonable to speculate that decrease of blood pressure might also play a role in amelioration of recognitive function.

## Conclusions

In conclusion, our present study demonstrates the anti-hypertensive and neuroprotective effects of Gao-Zi-Yao. Our data may provide basic evidence for clinical application of Gao-Zi-Yao in treatment with aged hypertension patients.

## Supplementary Information

**Additional file 1.** Western Supplement.

## Data Availability

All data generated or analyzed during this study are included in this published article.

## Abbreviations

SHR: Spontaneous hypertensive rats
AD: Alzheimer's disease
TCM: Traditional Chinese medicine
SBP: Systolic blood pressure
GluR1: Glutamate receptor A1
NMDAR: N-methyl-d-aspartate receptor
CaMKII: Calmodulin-dependent protein kinase II
p-CREB: Phosphorylated-cAMP responsive element-binding protein
DG: Dentate gyrus
