# Supplementary figures and images for: Gao-Zi-Yao improves learning and memory function in old spontaneous hypertensive rats
Source: BMC Complement Med Ther. 2022 May 28;22:147. doi: 10.1186/s12906-022-03630-0 (PMC9148521; doi:10.1186/s12906-022-03630-0)

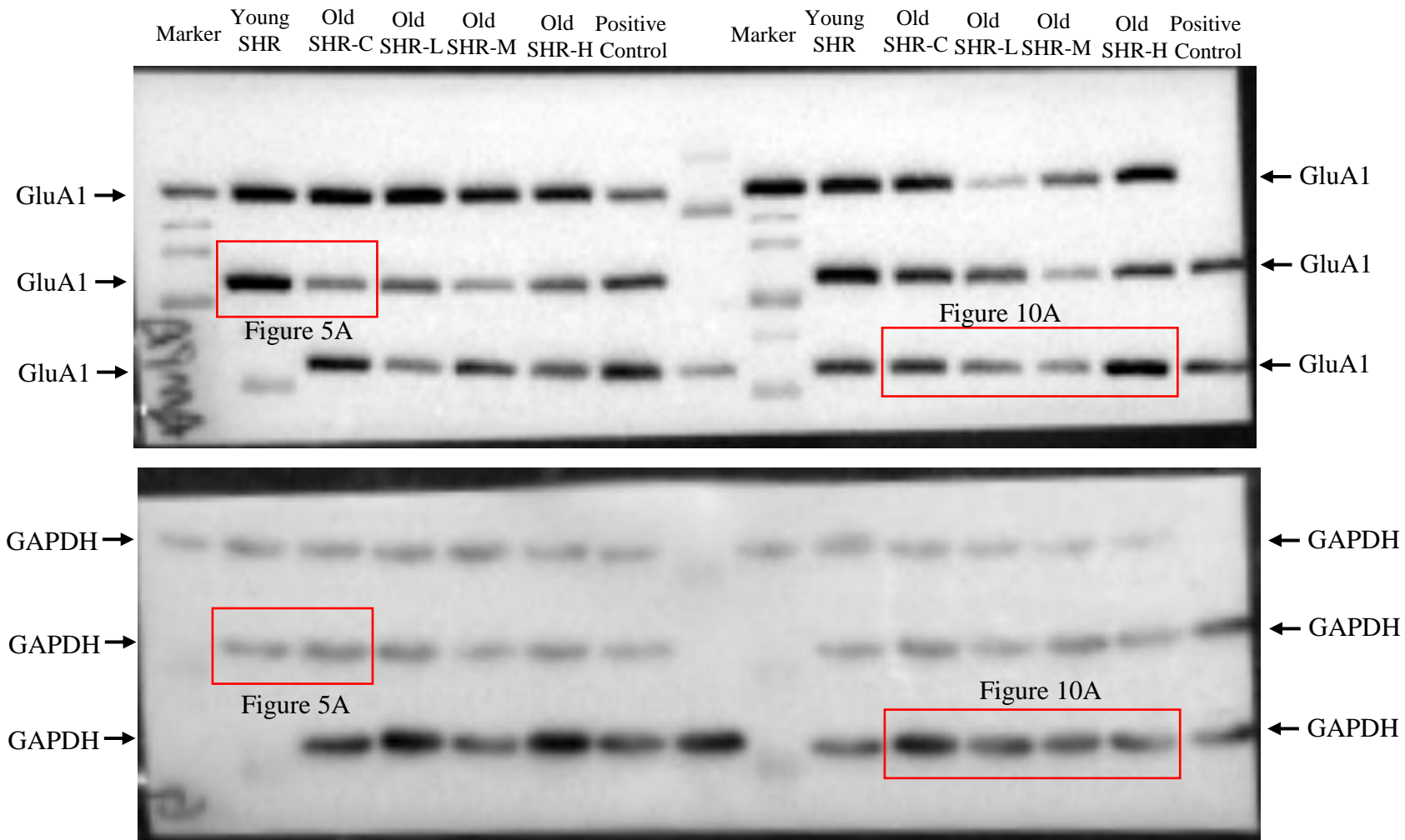

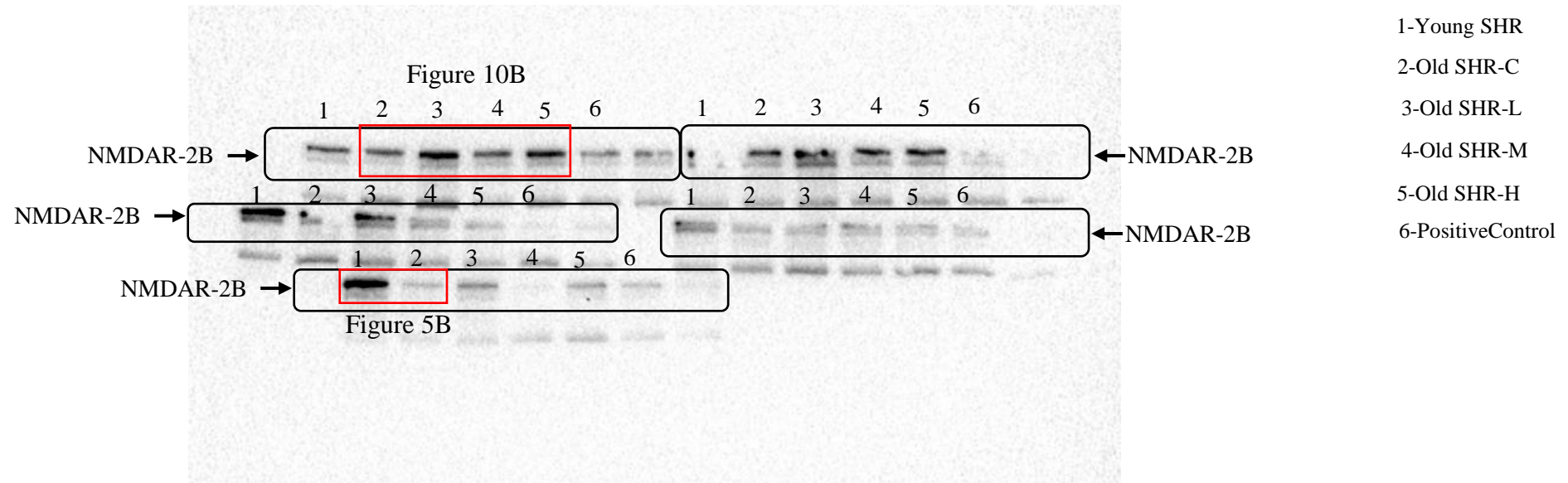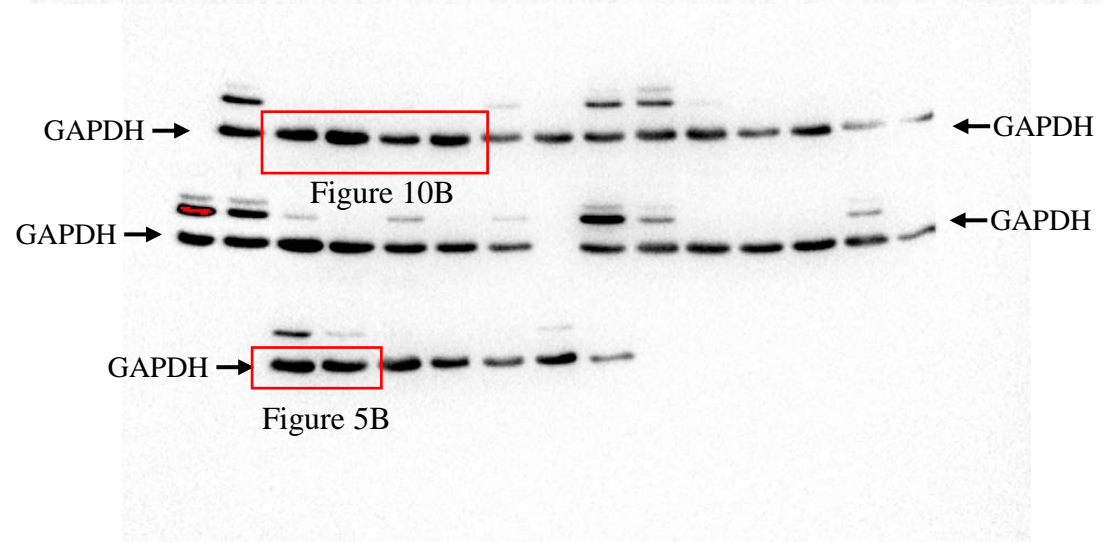

1-Young SHR  
 2-Old SHR-C  
 3-Old SHR-L  
 4-Old SHR-M  
 5-Old SHR-H  
 6-PositiveControl

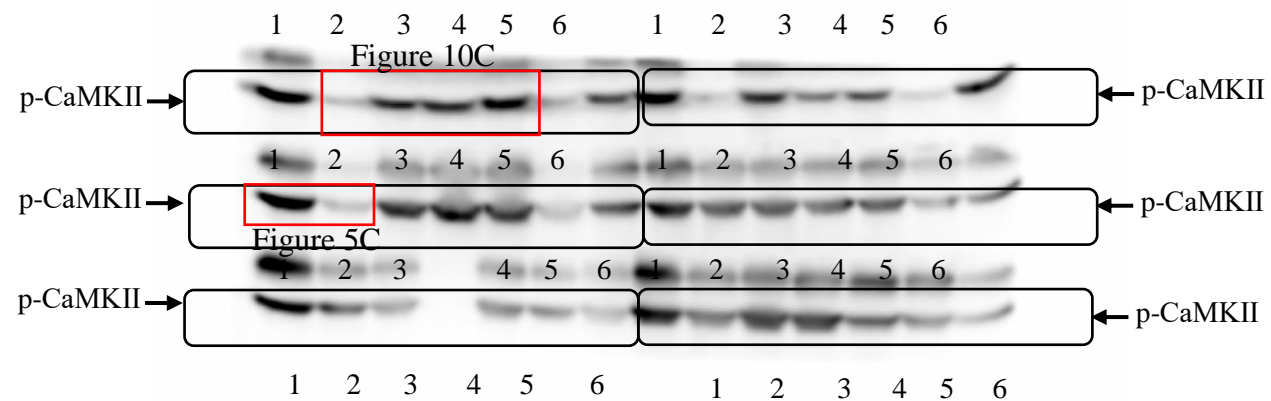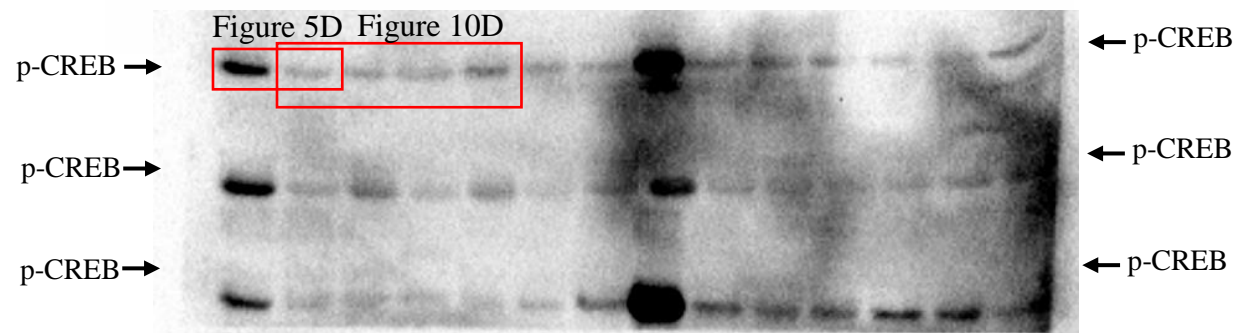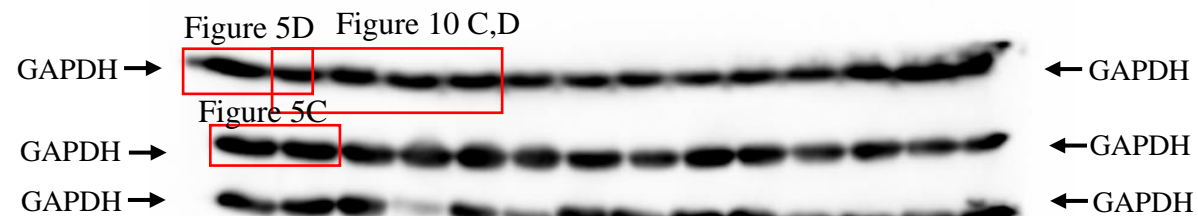

Supplement: Supplementary file 1 — Additional file 1. Western Supplement. [file 12906_2022_3630_MOESM1_ESM.pdf]
